# Extensive Residence in a Second Language Environment Modifies Perceptual Strategies for Suprasegmental Categorization

**DOI:** 10.1037/xlm0001246

**Published:** 2023-12

**Authors:** Katya Petrova, Kyle Jasmin, Kazuya Saito, Adam T. Tierney

**Affiliations:** 1 Department of Culture, Communication & Media, Institute of Education, University College London; 2 Department of Psychology, Royal Holloway University of London; 3 Department of Psychological Sciences, Birkbeck University of London

**Keywords:** prosody, cue weighting, length of residence, Mandarin, English

## Abstract

Languages differ in the importance of acoustic dimensions for speech categorization. This poses a potential challenge for second language (L2) learners, and the extent to which adult L2 learners can acquire new perceptual strategies for speech categorization remains unclear. This study investigated the effects of extensive English L2 immersion on speech perception strategies and dimension-selective-attention ability in native Mandarin speakers. Experienced first language (L1) Mandarin speakers (length of U.K. residence > 3 years) demonstrated more native-like weighting of cues to L2 suprasegmental categorization relative to inexperienced Mandarin speakers (length of residence < 1 year), weighting duration more highly. However, both the experienced and the inexperienced Mandarin speakers continued to weight duration less highly and pitch more highly during musical beat categorization and struggled to ignore pitch and selectively attend to amplitude in speech, relative to native English speakers. These results suggest that adult L2 experience can lead to retuning of perceptual strategies in specific contexts, but global acoustic salience is more resistant to change.

A design feature of speech that helps ensure comprehensibility despite variability in speakers, listeners, and environments is that multiple acoustic cues convey categories, enabling successful communication even if some cues are degraded. In other words, speech contrasts (both at the segmental and suprasegmental levels) are defined along multiple acoustic dimensions (Winter, 2014), with distributions that overlap along a single dimension (Hillenbrand et al., 1995; Holt & Lotto, 2010). In the case of segmental categories, for instance, voicing (as in “rapid” vs. “rabid”) is signaled by several acoustic dimensions, including duration (voice onset time) and pitch (Lisker, 1986). Similarly, suprasegmental features are cued by several acoustic dimensions, including pitch, duration, intensity, and spectral shape (Fear et al., 1995; Fonagy, 1978; Jasmin, Tierney, Obisah, & Holt, 2023).

This multiplicity of cues introduces a challenge, in that perceivers must decide on the extent to which each cue will drive their perception of a given phonological contrast. Learning the strength of evidence provided by each cue for the existence of a phonological category is particularly challenging when learning a second language (L2) in adulthood, because perceptual strategies learned for a first language (L1) may be suboptimal when applied to a new language. For example, for English /r/ and /l/, both the second and third formant (F2 and F3) onset frequencies contribute to category membership, but they differ in their perceptual weighting. Native English speakers rely on the onset frequency of the F3 significantly more than F2 when distinguishing between /r/ and /l/, because F3 most reliably signals the distinction between these two phonological categories (Espy-Wilson, 1992; Ingvalson et al., 2011; Iverson et al., 2003; Yamada & Tohkura, 1992). Native English speakers also make use of the onset frequency of F2, but it is a less reliable cue when categorizing /r/ versus /l/ because F2 frequency is subject to the influence of the surrounding phonetic context (Ingvalson et al., 2011). However, native Japanese speakers learning English as a L2 tend to rely much more on F2 than F3, because F3 is not a diagnostic dimension for consonant discrimination in Japanese (Iverson et al., 2003, 2005). To take another example, although English native speakers largely rely on spectral cues when categorizing English vowels, duration is relied upon more highly by learners of English speaking a variety of first languages, including Polish (Bogacka, 2004), Catalan (Cebrian, 2006), Spanish (Morrison, 2008), and Russian (Kondaurova & Francis, 2008).

Differences in perceptual strategies between nonnative and native speakers are not limited to segmental categorization but extend to suprasegmental categorization as well. In English, for example, many acoustic dimensions, including pitch, duration, amplitude, and vowel quality, help convey suprasegmental features such as lexical stress (Bolinger, 1958; Chrabaszcz et al., 2014; Fear et al., 1995; Fry, 1955; Gay, 1978; Morton & Jassem, 1965), contrastive sentence focus (Ladd & Morton, 1997), and phrase boundaries (Jusczyk et al., 1992). However, in Mandarin Chinese, syllabic pitch contours are essential for conveying lexical identity (Duanmu, 2007; Gandour, 1978, Howie, 1976), with comprehensibility greatly diminished in whispered speech (in which pitch is inaccessible; S. Liu & Samuel, 2004). The importance of pitch as a cue to lexical meaning in Mandarin affects the perceptual strategies Mandarin listeners use when learning English as a L2: Mandarin speakers favor pitch over other dimensions such as duration in their perception and production of word stress (Archibald, 1997; Juffs, 1990; Yu & Andruski, 2010, Y. Zhang et al., 2008), phrase boundaries (Jasmin, Sun, & Tierney, 2021), and sentence-level focus (Chen et al., 2001) compared to native English speakers.

What underlying mechanism accounts for the effects of L1 experience on L2 perceptual strategies? One possibility is that acoustic dimensions which are highly relevant to frequently encountered perceptual tasks acquire greater salience, that is, a bottom-up tendency to capture selective attention (Francis & Nusbaum, 2002; Gordon et al., 1993; Holt et al., 2018). Dimension-selective attention models of speech categorization strategies are supported by the finding that primary cues are down-weighted relative to secondary cues when listeners are given a second simultaneous task (Gordon et al., 1993). This suggests that attention to primary cues causes them to robustly drive categorization responses, but that they are not as influential when attentional resources are engaged elsewhere. According to dimension-selective attention models, Mandarin speakers, who have extensive experience using pitch cues to inform lexical category judgments while understanding everyday spoken language, may develop an overall bias to direct attention toward pitch and away from other suprasegmental cues such as duration or amplitude. Supporting this idea, Jasmin, Sun, and Tierney (2021) found that, compared to native English and Spanish speakers, Mandarin speakers had difficulty ignoring pitch and attending to amplitude in speech, even when explicitly asked to do so. This increased pitch salience may extend outside of speech to other domains such as music. Musical beats are conveyed by multiple acoustic dimensions, with strong beats linked to changes in pitch and duration (Ellis & Jones, 2009; Hannon et al., 2004). Musical beat perception, therefore, requires integration of information from multiple acoustic sources, and musical categorization strategies may be affected by dimensional salience. Jasmin, Sun, and Tierney (2021) found that Mandarin speakers showed increased pitch weighting during categorization of musical features (strong vs. weak beats), suggesting that language experience can have domain-general effects on dimensional salience.

Prior research, therefore, suggests that native language experience can have strong effects on perceptual strategies and dimensional salience. It remains unknown, however, whether these effects are limited to L1 experience in childhood, or whether experience with an L2 later in life can alter perceptual strategies and dimensional salience. Longer immersion in an L2 environment is associated with improved perceptual and production abilities in the target language (Aoyama et al., 2004; Flege et al., 1997, 1999; Trofimovich & Baker, 2006). More specifically, longer residency has been associated with more accurate production of vowels (Flege et al., 1997), consonants (Flege et al., 1995), and suprasegmental features (Trofimovich & Baker, 2006). However, some prior work has reported that perceptual strategies for L2 categorization did not change with immersion experience (Cebrian, 2006; Morrison, 2002); for example, native Japanese speakers’ weighting of the second versus third formants as cues to categorization of /r/ versus /l/ did not change as a function of their length of immersion experience (Ingvalson et al., 2011). Nevertheless, L2 perceptual strategies have been shown to change after short-term videogame training (Lim & Holt, 2011), and have been shown to change as adults age (Toscano & Lansing, 2019), suggesting that cue weights remain somewhat plastic even in adulthood.

To investigate whether adult L2 experience can affect perceptual strategies, we recruited experienced native Mandarin speakers with extensive immersion in an English-speaking environment (length of residence [LOR] > 3 years) and tested them on three tasks. In Task 1, they performed prosodic categorization on stimuli varying in pitch and duration patterns. In Task 2, they performed musical categorization on stimuli varying in pitch and duration. In Task 3, they listened to two-word phrases and were asked to selectively attend to either pitch or amplitude, ignoring the other dimension. We compared their performance to that of a native English group and a group of inexperienced Mandarin speakers (LOR < 1 year). (Data from the latter group were previously reported in Jasmin, Sun, & Tierney, 2021.) Analyzing Task 1 performance enabled us to ask whether longer residence in an English-speaking country can modulate Mandarin speakers’ L2 suprasegmental categorization strategies, decreasing reliance on pitch and/or increasing reliance on duration in categorizing English phrase boundaries (Jasmin, Sun, & Tierney, 2021).

If extensive adult L2 experience modifies L2 perceptual strategies, this could reflect either global changes in perceptual salience or context-specific learning. As mentioned above, evidence suggests that extensive childhood language experience can shape dimensional salience, leading to perceptual biases that transfer to other domains (including music). It is unknown, however, whether domain-general dimensional salience can also be shaped by adult L2 experience. Alternately, changes in L2 perceptual strategies after immersion in adulthood could reflect context-specific perceptual recalibration (Kleinschmidt & Jaeger, 2015). Listeners can fine-tune L1 perceptual strategies based on shifting evidence regarding the usefulness and reliability of cues in a particular context, a process which could also underlie successful L2 learning. For example, if intonation is an unreliable cue to a particular speaker’s intent, listeners will downweight intonational information, but only for that speaker (T. Roettger & Franke, 2019; T. B. Roettger & Rimland, 2020). Similarly, short-term changes in cue distribution can lead to down-weighting of the secondary cue and up-weighting of the primary cue for voicing (Idemaru & Holt, 2011), and the modified strategies do not generalize to a different place of articulation (Idemaru & Holt, 2014). If changing L2 perceptual strategies reflect context-specific statistical learning, then these changes may not transfer to other domains such as music, and baseline dimensional biases may be unchanged. We analyzed performance in Task 2 to investigate the specificity of the effect of extensive L2 experience by testing whether any shifts in perceptual biases due to L2 immersion transferred to the perceptual strategies used during categorization of musical beats cued by pitch and durational features. Finally, by analyzing Task 3, we investigated whether extensive L2 immersion can alter baseline dimensional salience by asking participants to either ignore pitch and attend to amplitude or to attend to pitch and ignore amplitude in a two-word phrase. If shifts in L2 perceptual strategies for the experienced Mandarin group reflect the development of context-specific strategies, we expected that long-term residents would continue to struggle to disengage attention from the pitch dimension.

## Method

### Participants

In total, 49 adult native speakers of Standard Mandarin residing in the United Kingdom and 50 adult native speakers of English were recruited for this study. The perceptual data of another language group consisting of 46 participants who were comparatively inexperienced L2 speakers of English will also be presented in this article. Data collection for them was completed at an earlier stage for a prior study (Jasmin, Sun, & Tierney, 2021). Only analyses comparing the experienced Mandarin residents to the other two groups will be presented here; for analyses comparing inexperienced Mandarin residents to native English speakers, see Jasmin, Sun, & Tierney, (2021). More detailed biographical information is presented separately for each group in the paragraphs that follow.

#### Experienced Mandarin Residents

Adult native speakers of Mandarin Chinese living in the United Kingdom (*n* = 49) with significant immersion experience were recruited for the present study (35 F, *M*_age_ = 29.8 years, *SD* = 7). Participants reported 3.8 mean years of musical training (*SD* = 4.6). In the current investigation, LOR was used as a key criterion for distinguishing between experienced (LOR > 3 years) and inexperienced (LOR < 1 year) participants. One weakness of LOR as a measure of L2 experience is that it does not reflect the frequency of L2 use, which varies widely across individuals immersed in an L2 environment, with some choosing to use only L1 rather than L2 (see Flege & Bohn, 2021). Thus, efforts were made to recruit only L2 individuals who mainly used English as their primary mode of communication (see below). As such, LOR was assumed to closely match how much the participants had engaged in L2 input (for similar methodological decisions, see Flege et al., 1995, 1996; Saito & Brajot, 2013; Trofimovich & Baker, 2006). The labeling of the groups here (LOR < 1 year for inexperienced; LOR > 3 years for experienced) was in line with Trofimovich and Baker (2006), that is, inexperienced (LOR < 1 year) and moderately/highly experienced (LOR > 3 years). Our decision also aligned with prior evidence showing that whereas various areas of adult L2 learners’ speech proficiency quickly change within the first year of immersion, a substantial amount of improvement can be observed only after they engage in an extensive period of immersion (e.g., Saito, 2015; Trofimovich & Baker, 2006; see also Munro et al., 2011 for a longitudinal examination of Chinese speakers’ L2 English phonetic development over 7 years).

Participants were recruited using multiple recruitment avenues. The research project was advertised to Mandarin speakers with at least 3 years of residency in the United Kingdom through postings on online community platforms and through word of mouth. A questionnaire adapted from Kachlicka et al. (2019) collected demographic information, measures of linguistic experience and language use, and musical training history. At the time of testing, Mandarin speakers’ LOR in the United Kingdom ranged from 3 to 29 years (*M* = 7.69, *SD* = 5.30). Participants had arrived in the country between the ages of 15 and 42 years (*M* = 21.69, *SD* = 4.56), and based on their age of arrival, they can be classified as late-onset Mandarin-English bilinguals (none of the participants grew up speaking English at home). Specifically, English was acquired as an L2 in a classroom context and on average participants reported 11.6 years of formal English instruction (*SD* = 4.7 years).

Participants were asked to evaluate their English oral proficiency on a 9-point scale (1 for *heavily-accented*, and 9 for *native-like*). All reported a high level of oral competence in their L2, indicating they used English in most of their daily activities (*M* = 60%). This represented a mean percentage use of 91.1%, 55.3%, and 32.3% where English was used as a main language of communication in professional, social, and home settings, respectively. Fifty-four (54%) percent of participants reported being exposed to a language other than English, but for the majority English was the most dominant L2. Some of the participants were also familiar with some regional dialects, but they were all native speakers of Standard Mandarin.

#### Inexperienced Mandarin Residents

A dataset including 46 native speakers of Mandarin (40 F, *M*_age_ = 23.5 years, *SD* = 1.9) was first reported in Jasmin, Sun, & Tierney (2021) and analyzed here as well. At the time of testing, they had only minimal immersion experience (*M*_LOR_ = 0.5 years, *SD* = 0.2 years) and had arrived in the country between the ages of 19 and 28 years (*M*_AOA_ = 22.9, *SD* = 1.8). They were all advanced users of English who had not previously lived in an English-speaking country apart from short holidays. At the time of testing, all participants were enrolled in undergraduate and graduate programs in U.K. universities. Participants in this group had received on average 13.7 years (*SD* = 2.3 years) of formal English instruction. They also self-reported 3.0 years (*SD* = 4.3) of musical training.

#### Native English Speakers

A dataset including 50 native speakers of English was recruited for the current study from the Prolific online recruitment platform (www.prolific.co). They were prescreened at an initial stage based on their native language background. Five of the participants were later excluded because they spoke a tone language as an L2 (Cantonese *n* = 3, Mandarin *n* = 2). This resulted in a total of 45 participants included in the final analysis (22 F, *M*_age_ = 28 years, *SD* = 4.1). They self-reported 0.7 (*SD* = 1.9) years of musical training. See Table 1 for a summary of the demographic characteristics of all participants.[Table tbl1]

None of the participants in the three groups reported any history of visual, auditory, or neurological impairments. Informed consent was obtained from all participants in compliance with an ethics protocol approved by the University College London Research Ethics Committee (applicable to the testing of the experienced Mandarin group), and Birkbeck, University of London (in relation to the testing of the other two groups). All participants were compensated for their time.

### General Experimental Procedure

The Gorilla Web Experiment Builder (www.gorilla.sc) was used for the creation and hosting of this study (Anwyl-Irvine et al., 2020), and all data were collected online. Before commencing the experiment, participants received instructions over email detailing the technical setup necessary for the smooth running of the experiment. They were asked to wear headphones. Participants were further encouraged to sit in a quiet room and minimize any distractions for the duration of the experiment. To ensure stimulus presentation was as similar as possible across participants, access to the testing environment was restricted only to computers running the Google Chrome browser. The experimental design followed closely the procedure adopted by Jasmin, Sun, and Tierney (2021) and all speech and auditory stimuli and test protocols were taken from the original study. The experiment was delivered in English to all three participant groups.

#### General Task Presentation

Participants were administered three tasks in total: a language task examining phrase boundary categorization (Task 1); a musical beat categorization task (Task 2); and a dimension-selective-attention task (Task 3). After these tasks were completed, the experienced Mandarin group completed a speech production task for an unrelated analysis that will not be reported further here. The first two tasks were presented in 20 alternating blocks (10 blocks per task), whereby a music beat categorization block was always followed by a phrase boundary categorization block to minimize boredom effects and ensure the continued engagement of participants. The remaining dimension-selective attention and spontaneous speech production tasks were presented once. The auditory tasks were preceded by a questionnaire designed to collect language background and music experience information about participants. The average running time for the experiment was approximately 60 min.

#### Prosodic Cue Weighting Task: Stimuli and Procedure

Participants performed prosodic categorization of a spoken phrase which could be judged as either having early or late phrase closure, cued by changes in syllable pitch and duration. The stimuli for this task were created by modifying natural speech recordings produced by a male native speaker of British English. Two sentences were recorded, each containing an initial set of six words that were identical lexically but differed in the location of a phrase boundary: “If Barbara gives up, the ship will be plundered” and “If Barbara gives up the ship, it will be plundered.” Both utterances instantiated a typical subordinate–main clause relationship with the first reading of the sentence reflecting early closure, and the second reading late closure. In their written form, the two grammatical structures were distinguished by the placement of the comma before *the ship*, or immediately after it. Only the first six words (which were identical lexically across both versions) were cropped from the recordings and used as a baseline for subsequent stimuli manipulation.

The STRAIGHT speech analysis and resynthesis software package (Kawahara & Irino, 2005) was used to morph the two phrases (see Jasmin, Dick, & Tierney, 2021 for details). F0 and duration were manipulated across five morphing levels recorded as percentage contributions from each of the two phrases. All other acoustic dimensions were held constant at an intermediate level, reflecting equal contributions from both recordings. The percentage values of the five morphing levels applied to the F0 and duration cues can be interpreted in the following manner. A 0% morphing level indicated pitch and duration patterns were entirely drawn from the early closure recording, while a 25% morphing rate signaled a greater contribution of acoustic information from the early closure recording. The 50% morphing condition carried equal contributions from both recordings. A 75% morphing rate indicated a greater contribution from the late closure recording. The F0 and duration patterns of the stimuli from the 100% morphing level were identical to the late closure recording. The fully crossed combinations of F0 and duration morphing levels resulted in a total of 25 unique stimuli.

Participants completed 250 trials (25 trials presented in blocks of 10) in which they listened to the stimulus and then had to select the utterance they thought they heard based on the location of the phrase boundary. The two phrase boundary interpretations were indicated by two buttons with contrasting comma positions. The first button read “If Barbara gives up, the ship” and it corresponded to a phrase boundary located in the middle of the excerpt. The comma on the second button appeared at the end of the phrase, as in “If Barbara gives up the ship,” indicating late closure.

Before proceeding to the testing session, participants were guided through an explanation of the orthographical difference between the two sentences and its significance in relation to how they were spoken. Participants were instructed to read both sentences aloud and they had the opportunity to hear an unmodified recording of each sentence 3 times. Finally, two practice trials with feedback were presented to familiarize participants with the testing procedure. The practice trials contained full unaltered recordings so that participants could hear the target phrases in context. During test trials, feedback was no longer present.

#### Prosodic Cue Weighting Task: Data Analysis

To analyze the relationship between LOR and cue weighting, we used logistic regression, a commonly used technique for investigating perceptual cue weighting (Schertz & Clare, 2020; Toscano & Lansing, 2019). Prosodic cue weighting was measured with a single mixed-effects logistic regression model (lme4 package; Bates et al., 2015) in R (R Core Team, 2022), with contrast difference coding (MASS package; Venables & Ripley, 2002) used to separately code fixed effects of the comparison between experienced and inexperienced Mandarin speakers (listed as “Mand_exp—Mand_inexp” in the regression tables) and the comparison between native English speakers and experienced Mandarin speakers (“Eng—Mand_exp”). The dependent variable was whether, for each trial, the participant indicated hearing the phrase boundary (i.e., the comma) after “up” (coded as 0) or after “ship” (coded as 1). The pitch (× 5 levels) and duration (× 5 levels) dimensions were centered, scaled, and entered as continuous linear predictor variables. Participant was included as a random intercept, plus random slopes for pitch level, duration level, and their interaction. The regression equation was (response ~ pitch_level × duration_level × group + (1 + pitch_level × duration_level |participant)). All graphical representations of the data were generated using the ggplot2 package in R (Wickham, 2016).

#### Music Cue Weighting Task: Stimuli and Procedure

This task assessed Mandarin speakers’ perceptual strategies in a nonverbal auditory domain in which pitch information contributes to categorization—music perception (Ellis & Jones, 2009; Palmer & Krumhansl, 1987; Prince, 2014; A. T. Tierney et al., 2011). Stimuli for this task were identical to those used in Jasmin, Sun, & Tierney (2021).

The stimuli for this task were generated using a custom MATrix LABratory (MATLAB; The MathWorks, Inc., Natick, Massachusetts) script. The tones were four-harmonic complex tones, with equal amplitude across harmonics, and a 15-ms on–off cosine ramp to avoid transients. The pitch and duration dimensions were manipulated across five levels and their arrangements signaled either a three-note grouping (STRONG weak weak STRONG weak weak) or a two-note grouping (STRONG weak STRONG weak STRONG weak). This was achieved by manipulating the pitch and/or duration of the first note in relation to the second and third notes. A higher pitch or longer duration of the first note, for instance, suggested the presence of a strong beat at that location.

Stimuli were presented as six-tone groupings repeated 3 times, such that participants always heard 18 tones on each trial. The pitch (× 5) and duration (× 5) levels were fully crossed so that their combinations resulted in 25 exemplars. For the pitch dimension, the tones either signaled a three-note grouping ([C# A A C# A A] and [B A A B A A]), did not indicate any note grouping ([A A A A A A]), or signaled a two-note grouping interpretation ([C# A C# A C# A] and [B A B A B A]). “A” corresponded to a frequency of 440 Hz, “B” was equal to 493.9 Hz, and “C#” was equal to 554.4 Hz. The five duration levels, expressed in milliseconds, either suggested a three-note grouping ([200 50 50 200 50 50] and [100 50 50 100 50 50]), did not point toward any note grouping ([50 50 50 50 50 50]), or suggested a grouping of two ([100 50 100 50 100 50] and [200 50 200 50 200 50]). On some trials, the pitch and duration combinations were consistent with one another, denoting the same grouping sequence, while on other trials they conflicted, signaling different interpretations of the note sequences.

All stimuli were presented once in each block, and participants had to categorize a total of 250 trials across ten 25-item blocks. At the beginning of the task, two practice trials were presented to exemplify a typical arrangement of a three-note and a two-note sequence. Participants were asked to categorize the grouping patterns as either a three-note or a two-note sequence.

#### Music Cue Weighting Task: Data Processing

Music cue weighting was measured in a similar manner to prosodic cue weighting, with a single mixed-effects logistic regression model, with contrast difference coding used to separately code fixed effects of the two group comparisons. The dependent variable was whether, for each trial, the participant indicated hearing “Groups of three (STRONG weak weak)” (coded as 0) or “Groups of two (STRONG weak)” (coded as 1). The pitch (× 5 levels) and duration (× 5 levels) dimensions were centered, scaled, and entered as continuous linear predictor variables. Participant was included as a random intercept, plus random slopes for pitch level, duration level, and their interaction. The regression equation was (response ∼ pitch_level × duration_level × group + (1 + pitch_level × duration_level |participant)).

#### Dimension-Selective Attention Task: Stimuli and Procedure

This test assessed the ability to direct selective attention to either pitch or amplitude, ignoring task-irrelevant variation in the unattended dimension. The stimulus set consisted of a two-word phrase “study music” extracted from recordings of the following sentences differing in the position of word emphasis: “Dave likes to STUDY music, but he doesn’t like to PLAY music” and “Dave likes to study MUSIC, but he doesn’t like to study HISTORY” (all-caps words indicate the location of contrastive focus).

A similar morphing procedure to the one described for the prosodic cue weighting task was used for the creation of these stimuli. However, for this task, pitch and amplitude were morphed across four levels cuing word emphasis either on the first word in the phrase “STUDY music” or the second word, as in “study MUSIC.” The morphing levels applied were expressed in percentages on a scale of 0%–100%, with 0% and 33% indicating greater acoustic contributions from the phrase with emphasis on the first word, and 67% and 100% indicating greater contribution from the recording with emphasis on the second word. Pitch and amplitude dimensions were fully crossed to create the stimulus space, resulting in 16 total stimuli. Each condition featured 48 trials (three presentations of the 16 phrase stimuli), amounting to 96 trials across two attention conditions (Attend Pitch and Attend Amplitude). The order of presentation of the two task conditions was identical for all participants, with the Attend Amplitude condition always coming first in the experiment followed by the Attend Pitch condition.

For each trial, a single stimulus was presented, and participants were asked to indicate which of the two words was either louder (Attend Amplitude condition) or pronounced in a higher pitch (Attend Pitch condition) by clicking one of two buttons labeled “1” or “2.” Trial-by-trial feedback was presented visually following each response. Participants saw a green check mark for correct responses and a red “x” mark if their responses were inaccurate.

### Dimension-Selective Attention Task: Data Processing

Data were summarized for each condition separately. Performance accuracy was computed as portion correct responses. Group comparisons were conducted comparing the experienced native Mandarin speakers to the inexperienced native Mandarin speakers and the native English speakers using Mann–Whitney tests, with correction for multiple comparisons. In addition, the influence of each dimension on participants’ responses in each attention condition was calculated using mixed-effects logistic regression. Two mixed-effects logistic regression models were conducted, one examining responses on the attention to pitch test and another examining responses on the attention to amplitude test, with contrast difference coding used to separately code the fixed effects of the two group comparisons. For the attention to amplitude test, the dependent variable was whether, for each trial, the participant indicated that the first word was louder (coded as 0) or the second word was louder (coded as 1). For the attention to pitch test, the dependent variable was whether, for each trial, the participant indicated that the first word was higher in pitch (coded as 0) or the second word was higher in pitch (coded as 1). The pitch (× 4 levels) and amplitude (× 4 levels) dimensions were centered, scaled, and entered as continuous linear predictor variables. Participant was included as a random intercept, plus random slopes for pitch level, amplitude level, and their interaction. The regression equation was (response ∼ pitch_level × amplitude_level × group + (1 + pitch_level × amplitude_level |participant)).

### Transparency and Openness

We report above how we determined our sample size, all data exclusions, all manipulations, and all measures in the study. All data and code needed to reproduce the analyses in this manuscript are available at https://osf.io/zq7sc/ (A. Tierney, 2023). This study’s design and its analysis were not preregistered. Study materials are not available online but can be made available upon request to the corresponding author.

## Results

**Figure 1 fig1:**
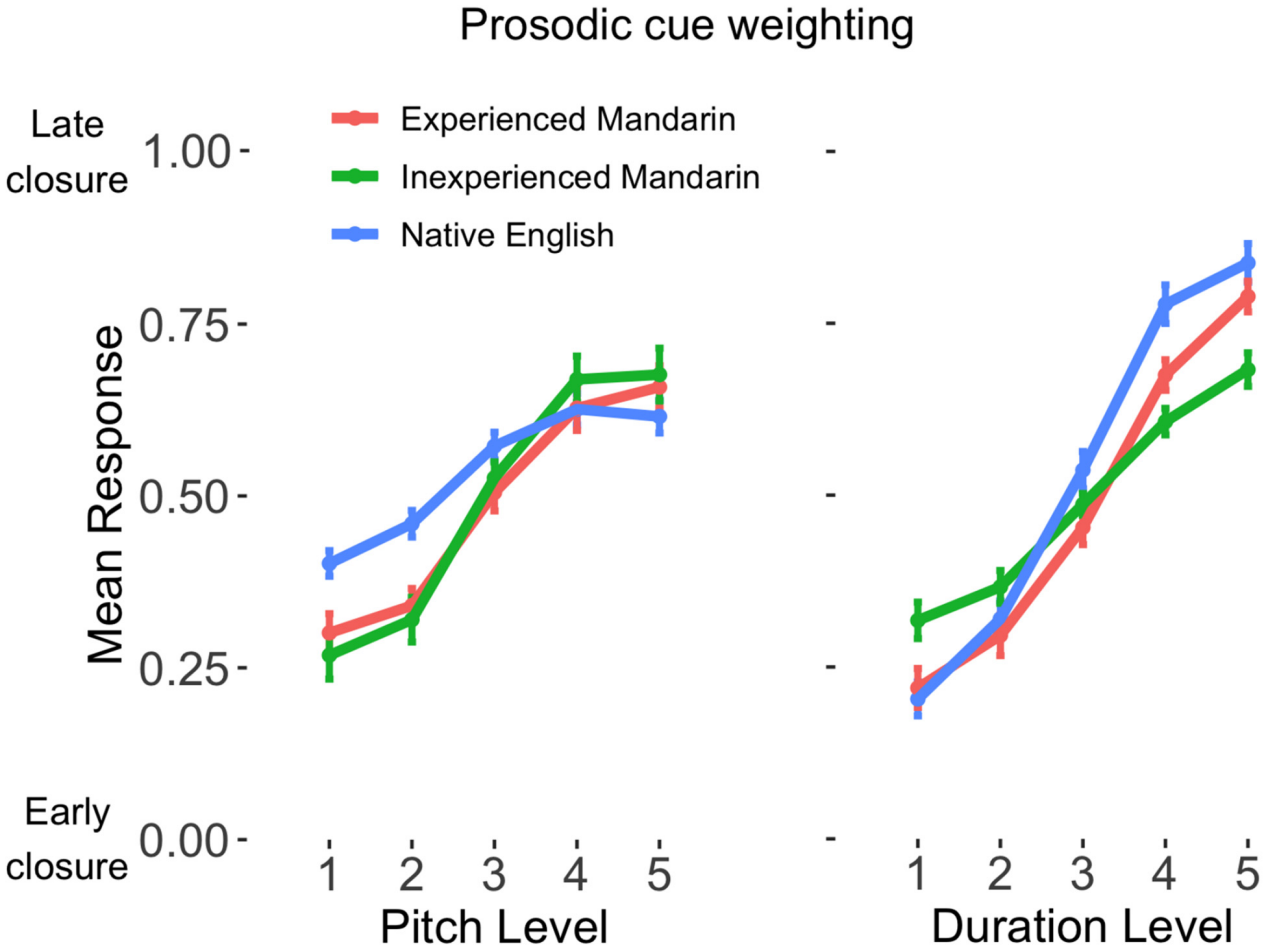
Mean Cross-Participant Prosody Categorization Response Across Pitch (Left) and Duration (Right) Levels *Note.* Errors bars indicate one standard error of the mean. See the online article for the color version of this figure.

Results of the mixed-effects logistic regression model analyzing prosody perception are summarized in Table 2. During prosody perception the experienced native Mandarin speakers weighted duration more highly compared to the inexperienced native Mandarin speakers (β = 0.68, *p* < .01). However, the experienced native Mandarin speakers’ pitch weighting was not significantly different to that of the inexperienced native Mandarin speakers (β = −0.20, *p* = .43). Compared to the experienced Mandarin speakers, the native English speakers weighted pitch less highly (β = −0.52, *p* = .04). See Figure 1 for plots of dependence of categorization on pitch and duration level for the prosody perception task across the three groups.[Table tbl2][Fig fig1]

**Figure 2 fig2:**
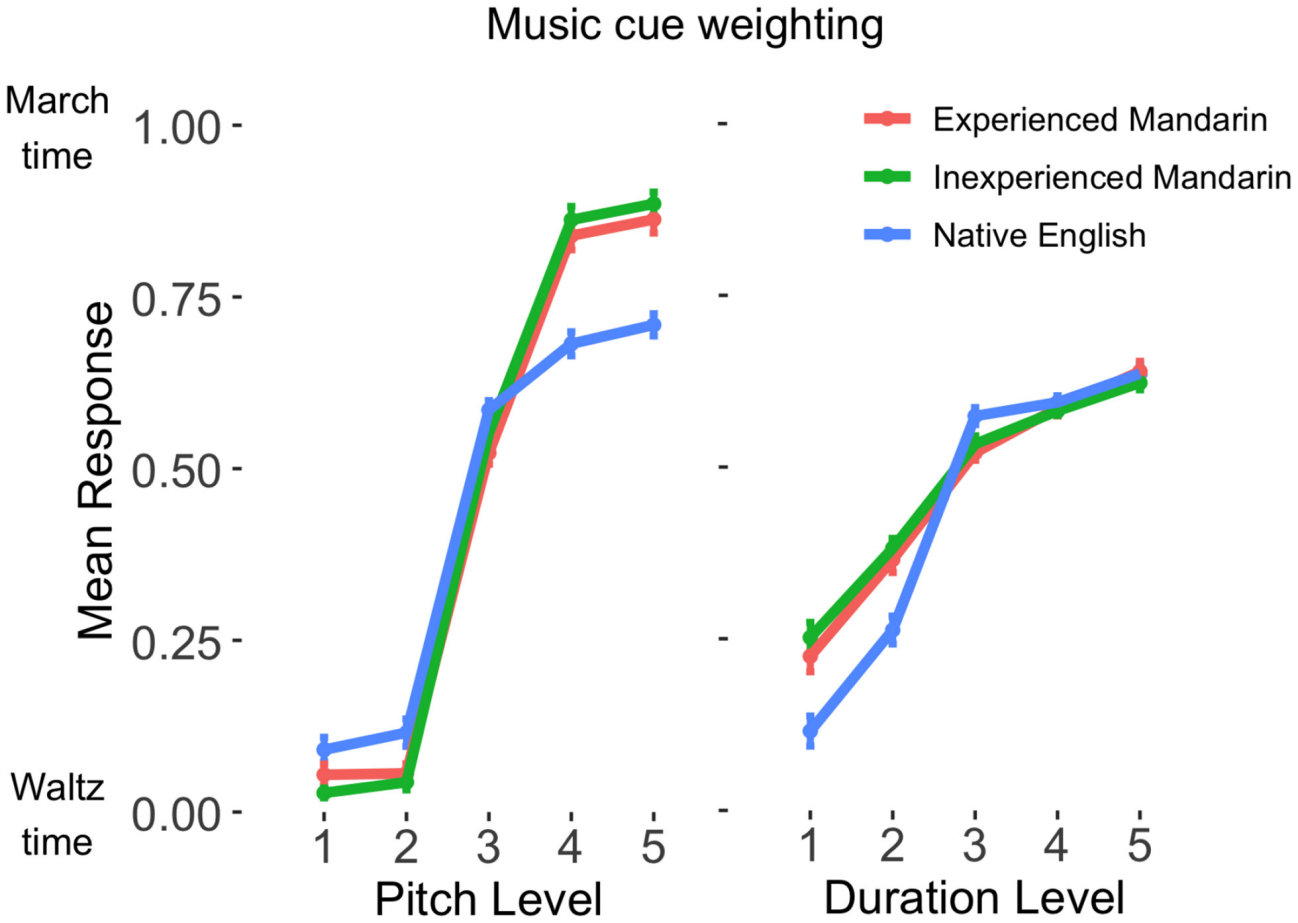
Mean Cross-Participant Musical Phrase Categorization Response Across Pitch (Left) and Duration (Right) Levels *Note.* Errors bars indicate one standard error of the mean. See the online article for the color version of this figure.

A different pattern of results was found for cue weighting during music perception, with inexperienced and experienced Mandarin speakers showing very similar perceptual strategies. Results of the mixed-effects logistic regression model analyzing music perception are summarized in Table 3. Duration was weighted similarly by inexperienced and experienced Mandarin speakers (β = 0.24, *p* = .29). Pitch was also weighted similarly by inexperienced and experienced Mandarin speakers (β = −0.22, *p* = .73). Compared to the experienced native Mandarin speakers, the native English speakers had lesser pitch weighting (β = −2.70, *p* < .01). There was, however, no significant difference in duration weighting between the experienced Mandarin and native English speakers (β = −0.29, *p* = .19). See Figure 2 for the dependence of categorization on pitch and duration level for the music perception task across the three groups.[Table tbl3][Fig fig2]

To directly test whether the difference between the inexperienced and experienced Mandarin speakers differed between the prosody and music perception tasks, we ran a mixed-effects logistic regression on the trial-wise data across both the music perception and prosody perception tasks, including data from the inexperienced and experienced Mandarin speakers. The regression equation was (response ∼ pitch_level × duration_level × group × task + (1 + pitch_level × duration_level × task|participant)). The results are displayed in Table 4. Neither the three-way interaction between pitch level, group, and task (β = 0.00, *p* = .99) nor the interaction between duration level, group, and task (β = −0.11, *p* = .09) was significant. Thus, it is not possible to conclude that the effect of experience was greater for the prosody compared to the music perception task, despite our finding of a significant effect of experience on cue weighting for prosody perception but not for music perception.[Table tbl4]

**Figure 3 fig3:**
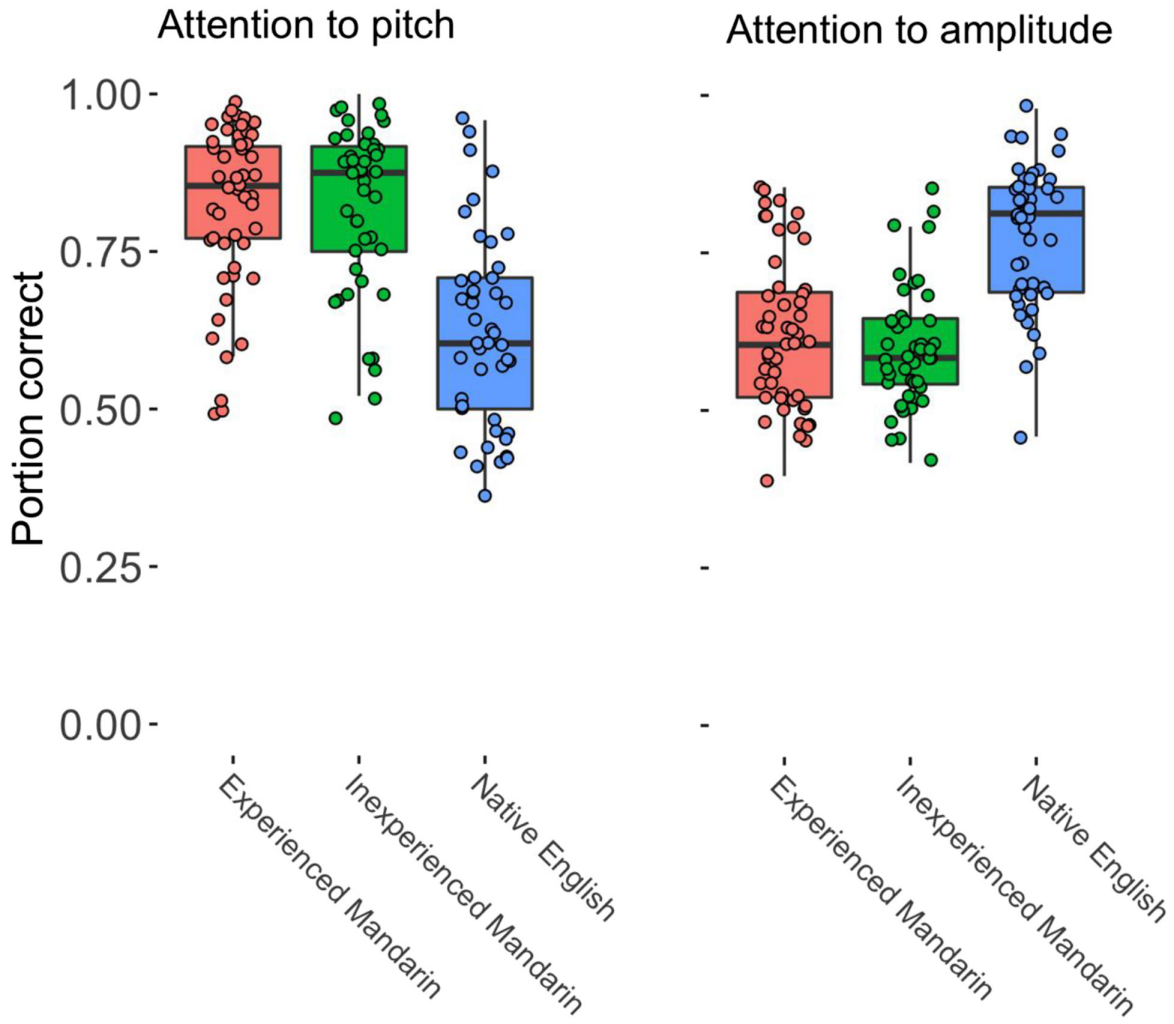
Dimension-Selective-Attention Performance in Attention to Pitch (Left) and Attention to Amplitude (Right) Conditions in Experienced L1 Mandarin Speakers, Inexperienced L1 Mandarin Speakers, and Native L1 English Speakers *Note.* The upper and lower boundaries of the box indicate the first and third quartiles. The upper and lower whiskers extend to the largest value within ±1.5 × interquartile range (IQR) of the edge of the box. L1 = first language; L2 = second language. See the online article for the color version of this figure.

The native Mandarin speaker groups performed similarly on the dimension-selective attention tests but showed a strikingly different pattern compared to native English speakers. On the attention to amplitude test, the inexperienced (*Mdn* = 58.3%) and experienced (*Mdn* = 60.4%) Mandarin speakers showed similar performance (*z* = −0.78, *p*_corrected_ = 1). On the attention to pitch test, the inexperienced (*Mdn* = 87.5%) and experienced (*Mdn* = 85.4%) Mandarin speakers once again showed similar performance (*z* = 0.34, *p*_corrected_ = 1.00). Relative to the experienced Mandarin speakers, the native English speakers performed better on the attention to amplitude test (*Mdn* = 81.3%; *z* = 5.49, *p*_corrected_ < .001) but worse on the attention to pitch test (*Mdn* = 60.4%; *z* = −5.51, *p*_corrected_ < .001). See Figure 3 for a plot of dimension-selective attention performance for inexperienced Mandarin speakers, experienced Mandarin speakers, and English speakers.[Fig fig3]

To ensure that our findings for the dimension-selective-attention data were not driven by our choice of nonparametric statistical tests, we reran all these analyses after transforming the data to approximate a normal distribution (rau transform). In addition, outliers of more than 2 *SD*s away from the mean were removed. We found that all the effects reported in the previous paragraph remained significant (*p*_corrected_ < .05). We also made use of this normalized data to test for difference in variance between the groups using Levene’s test for homogeneity of variance. We found that the inexperienced and experienced groups differed in variance for attention to amplitude performance, *F*(1, 89) = 13.5, *p*_corrected_ < .001; all other comparisons *p*_corrected_ > .1.

A follow-up analysis was conducted on the dimension-selective attention data to determine the relationship between the pitch and amplitude levels of the stimuli and participants’ responses. Results of the mixed-effects logistic regression models are summarized in Tables 5 and 6. For the attention to amplitude test (Table 5), the inexperienced and experienced Mandarin speakers had similar pitch weights (β = −0.07, *p* = .60) and amplitude weights (β = 0.17, *p* = .24). However, compared to the experienced Mandarin speakers, the native English speakers had lesser pitch weights (β = −0.95, *p* < .01) and greater amplitude weights (β = 1.03, *p* < .01). For the attention to pitch test (Table 6), the inexperienced and experienced Mandarin speakers had similar pitch weights (β = −0.17, *p* = .56) and amplitude weights (β = −0.06, *p* = .60). However, compared to the experienced Mandarin speakers, the native English speakers had lesser pitch weights (β = −1.55, *p* < .01) and greater amplitude weights (β = 0.23, *p* = .02) .[Table tbl5][Table tbl6]

We chose to analyze data from all participants who participated in the experiment, which could have included some participants who responded randomly. To explore whether random responding might have affected our results, we ran a supplementary analysis in which we removed any participant who did not show a significant relationship between both pitch cues and duration cues and categorization for either music or prosody perception. (Logistic regression was conducted on trial-wise data from each individual participant, and 0.05 was used as the threshold for significance.) One participant was removed from the inexperienced Mandarin speaker group, one from the experienced Mandarin speaker group, and one from the native English group. We have included in the online supplemental material regression tables for this subset of participants; the inclusion versus exclusion of these participants had no substantive effect on the article’s main results.

## Discussion

We find that L2 speech perceptual strategies can become more native-like after several years of immersion in an L2 environment. Specifically, after several years of residence in the United Kingdom, native Mandarin speakers weighted duration more highly as a cue to English phrase boundary categorization, relative to less experienced Mandarin speakers with less than a year of immersion experience. Thus, although learners of an L2 may begin by default with perceptual strategies borrowed from their L1, over time they can adapt to the distributional statistics of their new language environment.

Our finding that L2 suprasegmental perceptual strategies change after immersion contrasts with previous reports from research on segmental categorization. For example, both Morrison (2002) and Cebrian (2006) found no relationship between LOR and the tendency for L2 English learners to use duration more than native speakers when perceiving a vowel contrast in English. Similarly, Ingvalson et al. (2011) found that while longer LOR was linked to speech production intelligibility, it did not correlate with the relative weighting of F3 versus other cues to English /r-l/. One possible explanation for these contrasting results is that L2 suprasegmental perceptual strategies are more susceptible to change due to L2 exposure later in life, compared to segmental perceptual strategies. One way to test this explanation would be to compare the effect of LOR on suprasegmental and segmental perceptual strategies in the same participant population. There is some evidence for greater gains for suprasegmental compared to segmental dimensions of L2 pronunciation proficiency when learners engage in more input in naturalistic (Saito, 2015) and classroom settings (R. Zhang & Yuan, 2020).

We found that although longer residence was linked to up-weighting of duration for L2 prosody perception, there was no link between LOR and perceptual strategies during music perception. The experienced Mandarin speakers continued to show increased pitch weighting and decreased duration weighting relative to the native English speakers. This finding should be interpreted with caution, given that we did not find an interaction between group, task, and weighting of either pitch or duration; in other words, we cannot conclude that the effect of LOR on cue use differed between music and prosody perception, despite finding a significant effect in the latter case but not the former. Nevertheless, one possible explanation for the persistence of pitch-biased music perception strategies after immersion that could be investigated in future research relates to the difference in real-world relevance of language versus music perception. Perceptual ambiguity and inappropriate reliance on pitch-related cues when processing L2 input can lead to communication difficulties that can ultimately affect communication success. For instance, inappropriate manipulation of the acoustic correlates of lexical stress contributes to the perception of nonnative accents and can lead to lexical stress misidentification (Y. Zhang et al., 2008). Facing the possibilities of reduced comprehensibility and communication breakdowns may have pushed Mandarin speakers to develop listening strategies that resemble more closely those of native speakers, thus minimizing the likelihood of communication difficulties. However, changing their reliance on pitch information in contexts outside of the speech domain (as in the case of processing musical events) is less likely to confer practical benefits to Mandarin listeners. One way to test this explanation would be to investigate native Mandarin speakers who are also professional musicians who have come to an English-speaking environment to study music. We predict that Mandarin-speaking musicians studying music in an English-speaking environment will gradually begin to develop music perception strategies more similar to those of native English speakers, weighing duration more (and possibly pitch less) as a cue to musical features such as beats and phrases.

We find that native Mandarin speakers with several years of immersion experience continue to have extreme difficulties ignoring pitch and attending to another dimension (amplitude) when explicitly instructed to do so. Specifically, both the experienced and the inexperienced Mandarin speaker group showed near-ceiling performance on the attention to pitch condition of the dimension-selective attention test and near-floor performance on the attention to amplitude condition. Native English speakers, on the other hand, performed better than experienced Mandarin speakers on attention to amplitude but worse on attention to pitch. One implication of this finding is that increased pitch salience due to experience speaking a tone language as an L1 in childhood is highly stable and resistant to change later in life, even after years of immersion in a non-tone-language environment. In other words, there may be a sensitive period for modification of auditory dimensional salience due to experience, with large effects early in life but diminished or nonexistent effects later in life. The stability of tone-language-experience-driven increases in pitch salience is consistent with the finding that tone-language speakers show enhanced pitch tracking in the auditory brainstem (Chandrasekaran et al., 2007, 2009; Swaminathan et al., 2008) which extends to nonverbal stimuli (Bidelman et al., 2011; Krishnan et al., 2009). It remains unclear whether changes in domain-general dimensional salience could result from either more extensive or more intense adult L2 experience.

That LOR is linked to increased duration weighting during prosody perception but does not enhance the ability to explicitly ignore pitch and attend to amplitude in speech suggests that the shift in suprasegmental speech perception strategies reflects implicit rather than explicit mechanisms. In other words, if Mandarin speakers cannot intentionally direct attention away from the pitch and toward other dimensions, their change in L2 perceptual strategies after immersion experience must reflect mechanisms that do not draw upon attention. One possibility, for example, is that perceptual space is gradually warped by adult immersion experience, leading to enhanced discrimination of duration (Iverson & Kuhl, 1994; Iverson et al., 2003). Another possibility is that immersion experience could lead to implicit perceptual recalibration that is specific to a particular L2 speech context (Kleinschmidt & Jaeger, 2015).

On average, several years of residence were not sufficient for the development of native-like L2 speech perception strategies: native English speakers demonstrated weaker pitch weighting during prosody perception relative to experienced Mandarin speakers. However, there were very large individual differences among participants within both groups of Mandarin speakers. A portion of the inexperienced Mandarin speakers demonstrated native-like L2 perceptual strategies, while some of the experienced Mandarin speakers continued to not weigh duration very highly relative to pitch. Thus, native-like L2 suprasegmental perceptual strategies were achievable after a few years of immersion for some participants, but not universally. In fact, significant variance in perceptual strategies was present even in the native English speaker group. This is consistent with previous work showing individual variability in perceptual strategies in L2 learning contexts (Chandrasekaran et al., 2010; Idemaru et al., 2012; Kim et al., 2018; Kong & Edwards, 2015; Schertz et al., 2015) and among native speakers (Hazan & Rosen, 1991). An important direction for future study is to investigate the factors driving these individual differences in perceptual strategies. One possibility is that individual differences in the precision of dimension-specific auditory processing drive perceptual strategies, given that distributional variance has been linked to perceptual strategies during category learning (Holt & Lotto, 2006). Participants with a domain-general pitch deficit, for example, downweight pitch as a cue in suprasegmental categorization tasks (Jasmin et al., 2019).

Given that some, but not all, participants with extensive immersion experience achieved native-like L2 perceptual strategies, another topic for future study would be whether training can help individuals achieve native-like L2 perceptual strategies. Training focused on individual cues has been demonstrated to change perceptual strategies for L2 segmental categorization (Francis et al., 2000, 2008; Kondaurova & Francis, 2010; Ylinen et al., 2010) and lexical tone categorization (Chandrasekaran et al., 2016). Similar training could potentially also help L1 speakers of tone languages develop native-like L2 perceptual strategies for English prosody perception.

One notable limitation of the current study is that the results are based on a sample of university students (for the Mandarin inexperienced group) compared to a sample of highly immersed professionals for whom English is the dominant language in at least one of several communicative settings—work, home, or social life. Given the diversity of linguistic experience associated with immigrant communities, these results might not be generalizable to other long-term Mandarin residents with comparable length of immersion, but with more limited English-based interaction opportunities. Another limitation is our use of only two stimulus sets and our use of only one representative stimulus per domain (prosody and music). A promising future direction would be to investigate how perceptual strategies for other prosodic features change with LOR in tone-language speakers. For example, the weighting of acoustic cues to English word stress differs between L1 tone-language speakers and native speakers of English (Wang, 2008; Yu & Andruski, 2010; Y. Zhang & Francis, 2010). Similarly, prosodic cue production differs between tone-language speakers and native speakers of English, but it remains unclear how cue production relates to LOR. Future work should also examine musical and prosodic perceptual strategies using a wider variety of stimuli to confirm that the differences in musical perceptual strategies between Mandarin and English speakers and the effects of LOR on prosodic perceptual strategies generalize to prosody and music perception in general rather than being limited to the specific stimuli used here.

In conclusion, we find that adult immersive L2 experience can alter the way individuals combine information across acoustic dimensions during L2 speech perception. However, the effects of L1 experience on dimensional salience and nonverbal perceptual strategies are more resistant to change. In adulthood, therefore, an individual’s perceptual strategies reflect biases laid down by developmental experience and flexible fine-tuning due to more recent learning.

## Supplementary Material

10.1037/xlm0001246.supp

## Figures and Tables

**Table 1 tbl1:** Means, Standard Deviations (in Parentheses), and Ranges for Participant Variables by Group

	LOR bands					
	Inexperienced residents		Experienced residents		Native English	
	(*N* = 46)		(*N* = 49)		(*N* = 45)	
Variables	*M* (*SD*)	Range	*M* (*SD*)	Range	*M* (*SD*)	Range
Age (years)	23.5 (1.9)	20–29	29.8 (7.0)	20–58	28 (4.1)	21–36
LOR (years)	0.5 (0.2)	0.4–1	7.7 (5.4)	3–29	—	—
Gender	40 F, 6 M		35 F, 14 M		22 F, 22 M, 1 other	
Music training	3.0 (4.3)	0–20	3.8 (4.6)	0–21	0.7 (1.9)	0–10
*Note.* LOR = length of residence; F = female; M = male.						

**Table 2 tbl2:** Regression Model Predicting Prosody Categorization Responses

Predictor	Estimate	*SE*	*z*	*p*
Intercept	0.0142	0.0680	0.21	.83
Pitch level	1.0034	0.1032	9.72	<.01
Duration level	1.4010	0.0881	15.89	<.01
Group (Mand_exp—Mand_inexp)	−0.0710	0.1649	−0.43	.67
Group (Eng—Mand_exp)	0.3207	0.1659	1.93	.05
Pitch:Duration	0.0278	0.0241	1.15	.25
Pitch:Group (Mand_exp—Mand_inexp)	−0.1974	0.2508	−0.79	.43
Pitch:Group (Eng—Mand_exp)	−0.5197	0.2508	−2.07	.04
Duration:Group (Mand_exp—Mand_inexp)	0.6802	0.2127	3.20	<.01
Duration:Group (Eng—Mand_exp)	0.1185	0.2151	0.55	.58
Pitch:Duration:Group (Mand_exp—Mand_inexp)	0.0219	0.0556	0.39	.69
Pitch:Duration:Group (Eng—Mand_exp)	−0.0761	0.0556	−1.37	.17

**Table 3 tbl3:** Regression Model Predicting Music Categorization Responses

Predictor	Estimate	*SE*	*z*	*p*
Intercept	0.1987	0.0700	2.84	<.01
Pitch level	5.1350	0.2649	19.39	<.01
Duration level	2.3480	0.0950	24.71	<.01
Group (Mand_exp—Mand_inexp)	−0.0026	0.1696	−0.02	.99
Group (Eng—Mand_exp)	−0.2039	0.1673	−1.22	.22
Pitch:Duration	1.8487	0.1315	14.06	<.01
Pitch:Group (Mand_exp—Mand_inexp)	−0.2215	0.6302	−0.35	.73
Pitch:Group (Eng—Mand_exp)	−2.7010	0.6215	−4.35	<.01
Duration:Group (Mand_exp—Mand_inexp)	0.2392	0.2255	1.06	.29
Duration:Group (Eng—Mand_exp)	−0.2910	0.2241	−1.30	.19
Pitch:Duration:Group (Mand_exp—Mand_inexp)	0.2151	0.3117	0.69	.49
Pitch:Duration:Group (Eng—Mand_exp)	0.1518	0.3071	0.49	.62

**Table 4 tbl4:** Regression Model Predicting Prosody and Music Categorization Across Inexperienced and Experienced Mandarin Speaker Groups

Predictor	Estimate	*SE*	*z*	*p*
Intercept	0.09	0.06	1.46	.14
Pitch level	3.75	0.21	18.08	<.01
Duration level	1.84	0.08	22.33	<.01
Group	−0.02	0.06	−0.29	.77
Task	0.18	0.06	2.73	<.01
Pitch:Duration	0.93	0.09	10.48	<.01
Pitch:Group	−0.11	0.20	−0.54	.59
Duration:Group	0.23	0.08	2.81	<.01
Pitch:Task	2.53	0.20	12.49	<.01
Duration:Task	0.60	0.07	8.95	<.01
Group:Task	0.02	0.06	0.31	.76
Pitch:Duration:Group	0.07	0.08	0.82	.41
Pitch:Duration:Task	0.88	0.09	10.23	<.01
Pitch:Group:Task	0.00	0.20	−0.01	.99
Duration:Group:Task	−0.11	0.07	−1.70	.09
Pitch:Duration:Group:Task	0.05	0.08	0.60	.55

**Table 5 tbl5:** Regression Model Predicting Trial-by-Trial Attention to Amplitude Responses

Predictor	Estimate	*SE*	*z*	*p*
Intercept	0.1222	0.0338	3.61	<.01
Pitch level	0.4140	0.0549	7.54	<.01
Amplitude level	0.9784	0.0629	15.55	<.01
Group (Mand_exp—Mand_inexp)	0.0082	0.0770	0.11	.91
Group (Eng—Mand_exp)	0.1952	0.0826	2.36	.02
Pitch:Amplitude	0.0531	0.0382	1.39	.16
Pitch:Group (Mand_exp—Mand_inexp)	−0.0682	0.1306	−0.52	.60
Pitch:Group (Eng—Mand_exp)	−0.9521	0.1339	−7.11	<.01
Amplitude:Group (Mand_exp—Mand_inexp)	0.1699	0.1447	1.17	.24
Amplitude:Group (Eng—Mand_exp)	1.0343	0.1541	6.71	<.01
Pitch:Amplitude:Group (Mand_exp—Mand_inexp)	−0.0302	0.0811	−0.37	.71
Pitch:Amplitude:Group (Eng—Mand_exp)	0.0984	0.0921	1.07	.29

**Table 6 tbl6:** Regression Model Predicting Trial-by-Trial Attention to Pitch Responses

Predictor	Estimate	*SE*	*z*	*p*
Intercept	−0.0136	0.0445	−0.31	.76
Pitch level	1.9064	0.1225	15.57	<.01
Amplitude level	0.2268	0.0443	5.12	<.01
Group (Mand_exp—Mand_inexp)	0.0250	0.1069	0.23	.81
Group (Eng—Mand_exp)	−0.1359	0.1009	−1.35	.18
Pitch:Amplitude	0.0074	0.0554	0.13	.89
Pitch:Group (Mand_exp—Mand_inexp)	−0.1705	0.2945	−0.58	.56
Pitch:Group (Eng—Mand_exp)	−1.5521	0.2857	−5.43	<.01
Amplitude:Group (Mand_exp—Mand_inexp)	−0.0551	0.1063	−0.52	.60
Amplitude:Group (Eng—Mand_exp)	0.2331	0.1005	2.32	.02
Pitch:Amplitude:Group (Mand_exp—Mand_inexp)	0.1374	0.1107	1.24	.21
Pitch:Amplitude:Group (Eng—Mand_exp)	−0.0289	0.1008	−0.29	.77
