# Supplementary material for: Extensive Residence in a Second Language Environment Modifies Perceptual Strategies for Suprasegmental Categorization
Source: J Exp Psychol Learn Mem Cogn. 2023 Dec;49(12):1943–55. doi: 10.1037/xlm0001246 (PMC10734206; doi:10.1037/xlm0001246)
Supplement: Supplementary file 1 [file xlm0001246_sm.docx]

**Table 1**

*Means, standard deviations (in parentheses), and ranges for participant variables by group for the subset of participants who passed the compliance screening.*

| LOR Bands | | | | | | |
| --- | --- | --- | --- | --- | --- | --- |
|  | Inexperienced residents | | Experienced residents | | Native English | |
|  | (N = 45) |  | (N = 48) |  | (N = 44) |  |
| Variables | M (SD) | Range | M (SD) | Range | M (SD) | Range |
| Age (years) | 23.5 (1.9) | 20 - 29 | 29.9 (7.1) | 20 - 58 | 28 (4.1) | 21 - 36 |
| LOR (years) | 0.5 (0.2) | 0.4 - 1 | 7.7 (5.3) | 3 - 29 | - | - |
| Gender | 39 F, 6 M |  | 34 F, 14 M |  | 22 F, 21 M, 1 other |  |
| Music training | 2.9 (4.4) | 0 - 20 | 3.9 (4.6) | 0 - 21 | 0.7 (1.9) | 0 - 10 |

**Table S2**

*Regression model predicting prosody categorization for the subset of participants who passed the compliance screening.*

|  | Est | S.E. | z | p |
| --- | --- | --- | --- | --- |
| (Intercept) | 0.0400 | 0.0671 | 0.60 | 0.55 |
| Pitch level | 1.0169 | 0.1052 | 9.67 | <0.01 |
| Duration level | 1.4267 | 0.0886 | 16.10 | <0.01 |
| Group (Mand_exp – Mand_inexp) | -0.1037 | 0.1627 | -0.64 | 0.52 |
| Group (Eng – Mand_exp) | 0.3398 | 0.1638 | 2.07 | 0.04 |
| Pitch:Duration | 0.0308 | 0.0246 | 1.25 | 0.21 |
| Pitch:Group (Mand_exp – Mand_inexp) | -0.2208 | 0.2551 | -0.87 | 0.39 |
| Pitch:Group (Eng – Mand_exp) | -0.5168 | 0.2553 | -2.02 | 0.04 |
| Duration:Group (Mand_exp – Mand_inexp) | 0.6870 | 0.2143 | 3.21 | <0.01 |
| Duration:Group (Eng – Mand_exp) | 0.1355 | 0.2165 | 0.63 | 0.53 |
| Pitch:Duration:Group (Mand_exp – Mand_inexp) | 0.0255 | 0.0569 | 0.45 | 0.65 |
| Pitch:Duration:Group (Eng – Mand_exp) | -0.0776 | 0.0571 | -1.36 | 0.17 |

**Table S3**

*Regression model predicting music for the subset of participants who passed the compliance screening.*

|  | Est | S.E. | z | p |
| --- | --- | --- | --- | --- |
| (Intercept) | 0.1966 | 0.0714 | 2.75 | <0.01 |
| Pitch level | 5.1768 | 0.2631 | 19.67 | <0.01 |
| Duration level | 2.3821 | 0.0928 | 25.68 | <0.01 |
| Group (Mand_exp – Mand_inexp) | 0.0241 | 0.1731 | 0.14 | 0.89 |
| Group (Eng – Mand_exp) | -0.2111 | 0.1709 | -1.23 | 0.22 |
| Pitch:Duration | 1.8691 | 0.1317 | 14.19 | <0.01 |
| Pitch:Group (Mand_exp – Mand_inexp) | -0.0051 | 0.6289 | -0.01 | 0.99 |
| Pitch:Group (Eng – Mand_exp) | -2.7676 | 0.6201 | -4.46 | <0.01 |
| Duration:Group (Mand_exp – Mand_inexp) | 0.3047 | 0.2198 | 1.39 | 0.17 |
| Duration:Group (Eng – Mand_exp) | -0.3064 | 0.2186 | -1.40 | 0.16 |
| Pitch:Duration:Group (Mand_exp – Mand_inexp) | 0.2888 | 0.3127 | 0.92 | 0.36 |
| Pitch:Duration:Group (Eng – Mand_exp) | 0.1528 | 0.3086 | 0.50 | 0.62 |

**Table S4**

*Regression model predicting prosody and music categorization across inexperienced and experienced Mandarin speaker groups for the subset of participants who passed the compliance screening.*

|  | Est | S.E. | z | p |
| --- | --- | --- | --- | --- |
| (Intercept) | 0.10 | 0.07 | 1.60 | 0.11 |
| Pitch level | 3.77 | 0.21 | 18.22 | <0.01 |
| Duration level | 1.87 | 0.08 | 23.07 | <0.01 |
| Group | -0.02 | 0.06 | -0.30 | 0.76 |
| Experiment | 0.16 | 0.06 | 2.51 | 0.01 |
| Pitch:Duration | 0.93 | 0.09 | 10.50 | <0.01 |
| Pitch:Group | -0.06 | 0.20 | -0.27 | 0.78 |
| Duration:Group | 0.25 | 0.08 | 3.12 | <0.01 |
| Pitch:Experiment | 2.53 | 0.20 | 12.58 | <0.01 |
| Duration:Experiment | 0.61 | 0.07 | 8.92 | <0.01 |
| Group:Experiment | 0.04 | 0.06 | 0.55 | 0.58 |
| Pitch:Duration:Group | 0.09 | 0.08 | 1.10 | 0.27 |
| Pitch:Duration:Experiment | 0.88 | 0.09 | 10.19 | <0.01 |
| Pitch:Group:Experiment | 0.06 | 0.20 | 0.33 | 0.74 |
| Duration:Group:Experiment | -0.10 | 0.07 | -1.44 | 0.15 |
| Pitch:Duration:Group:Experiment | 0.07 | 0.08 | 0.87 | 0.39 |

**Table S5**

*Regression model predicting trial-by-trial attention to amplitude responses for the subset of participants who passed the compliance screening.*

|  | Est | S.E. | z | p |
| --- | --- | --- | --- | --- |
| (Intercept) | 0.1253 | 0.0337 | 3.72 | <0.01 |
| Pitch level | 0.4125 | 0.0559 | 7.38 | <0.01 |
| Amplitude level | 0.9911 | 0.0634 | 15.62 | <0.01 |
| Group (Mand_exp – Mand_inexp) | 0.0266 | 0.0764 | 0.35 | 0.73 |
| Group (Eng – Mand_exp) | 0.2111 | 0.0825 | 2.56 | 0.01 |
| Pitch:Amplitude | 0.0419 | 0.0383 | 1.09 | 0.27 |
| Pitch:Group (Mand_exp – Mand_inexp) | -0.0452 | 0.1328 | -0.34 | 0.73 |
| Pitch:Group (Eng – Mand_exp) | -0.9775 | 0.1364 | -7.16 | <0.01 |
| Amplitude:Group (Mand_exp – Mand_inexp) | 0.1771 | 0.1455 | 1.22 | 0.22 |
| Amplitude:Group (Eng – Mand_exp) | 1.0712 | 0.1555 | 6.89 | <0.01 |
| Pitch:Amplitude:Group (Mand_exp – Mand_inexp) | -0.0369 | 0.0807 | -0.46 | 0.65 |
| Pitch:Amplitude:Group (Eng – Mand_exp) | 0.1173 | 0.0927 | 1.27 | 0.21 |

**Table S6**

*Regression model predicting trial-by-trial attention to pitch responses for the subset of participants who passed the compliance screening.*

|  | Est | S.E. | z | p |
| --- | --- | --- | --- | --- |
| (Intercept) | -0.0239 | 0.0431 | -0.55 | 0.58 |
| Pitch level | 1.9147 | 0.1243 | 15.4 | <0.01 |
| Amplitude level | 0.2394 | 0.0442 | 5.41 | <0.01 |
| Group (Mand_exp – Mand_inexp) | 0.0544 | 0.1029 | 0.53 | 0.60 |
| Group (Eng – Mand_exp) | -0.1829 | 0.0969 | -1.89 | 0.06 |
| Pitch:Amplitude | 0.0171 | 0.0563 | 0.3 | 0.76 |
| Pitch:Group (Mand_exp – Mand_inexp) | -0.1163 | 0.2987 | -0.39 | 0.70 |
| Pitch:Group (Eng – Mand_exp) | -1.5629 | 0.2901 | -5.39 | <0.01 |
| Amplitude:Group (Mand_exp – Mand_inexp) | -0.0644 | 0.1059 | -0.61 | 0.54 |
| Amplitude:Group (Eng – Mand_exp) | 0.2466 | 0.1003 | 2.46 | 0.01 |
| Pitch:Amplitude:Group (Mand_exp – Mand_inexp) | 0.1661 | 0.1123 | 1.48 | 0.14 |
| Pitch:Amplitude:Group (Eng – Mand_exp) | -0.05 | 0.1027 | -0.49 | 0.63 |
